# Embracing change: Navigating menopause with the help of mobile health apps in Germany

**DOI:** 10.1016/j.cpnec.2025.100320

**Published:** 2025-09-25

**Authors:** Klara Greffin, Marlene Muehlmann, Anna Pauleikhoff, Bita Stelling, Holger Muehlan, Michael Stach, Silke Schmidt, Samuel Tomczyk

**Affiliations:** a University of Greifswald, Institute of Psychology, Department Health and Prevention, Germany; b Health & Medical University Erfurt, Medical Department, Division Medical Psychology, Germany; c Institute of Clinical Epidemiology and Biometry, University of Würzburg, Würzburg, Germany

**Keywords:** Digital health application, mHealth, Telemedicine, Menopause, Climacteric, Evaluation, App review, Women's health, Smartphone apps

## Abstract

**Background:**

Mobile health applications (mHealth apps) have shown promise as potential solutions for mitigating the negative health impacts of menopause. However, there is a lack of standardized expert and user ratings regarding the quality of these apps, making it difficult to provide evidence-based recommendations. Therefore, this app review aimed to summarize the currently available mHealth apps for menopause in Germany. Subsequently, the identified apps were evaluated, and their quality was assessed.

**Methods:**

A web crawler was used to search for mHealth apps related to the predefined search criteria, and information for all included apps was extracted (year of search: 2021). In the second step, healthcare providers and individuals experiencing menopause tested and evaluated the apps using the German version of the Mobile Application Rating Scale (MARS-G).

**Results:**

The initial search yielded 6917 apps. Of these, 6879 were excluded, 32 duplicates were removed, three were available only in English, and one was designed for a specific drug treatment. The two remaining apps, Femilog and Mimeno, received low expert ratings (*n* = 2) for engagement, therapeutic gain, and subjective quality. In the user ratings (*n* = 4), both apps received average scores across all MARS-G scales.

**Conclusion:**

This app review identified two mHealth apps for menopause in German that met the inclusion criteria. Both apps were found to have poor perceived therapeutic gain and received mixed ratings and recommendations from experts and users. Further studies on user experience and efficacy are necessary to make evidence-based recommendations regarding mHealth apps for individuals experiencing menopause.

## Highlights

• Only two apps met the inclusion criteria – highlighting a lack of evidence-based mHealth options for menopause.
• User and expert ratings of the apps differed, showing the value of multiple perspectives in app evaluation.
• Many menopause apps lack scientific validation, raising concerns about safety, effectiveness, and user suitability.

Menopause is a natural biological process that marks the end of an individual's reproductive years and is therefore connected to human ageing [1,2]. From a clinical perspective, experts have defined menopause as “the anchor point that is defined after 12 months of amenorrhea following the final menstrual period (FMP), which reflects a near complete but natural diminution of ovarian hormone secretion” [1]. On average, menopause begins around 51 years of age [3,4], although with considerable variety in the population [5].

Individuals who experience menopause often face several physical and emotional challenges: Insomnia was reported by about 38 % of over 12,000 participants experiencing menopause between the ages of 40 and 55 years [6]. Vasomotor symptoms like hot flashes often begin in the menopausal transition period between a noticeable monotropic follicle stimulating hormone (FSH) rise and the FMP, and can last up to four years after menopause [7,8]. These symptoms can also be connected to negative mood or depressive symptoms [[9], [10], [11]], particularly in proximity to the FMP, but with a decrease in depressive symptoms after two to eight years post-FMP [12,13]. Severe vasomotor symptoms may impair personal and social life, sexual health, psychological well-being, and the ability to work. These symptoms often remain unrecognized and unaddressed, and thus, may affect quality of life negatively [[14], [15], [16], [17]]. Many studies further report significant associations between menopausal transition and poor mental health and cognitive performance [13,14,16].

Consequently, menopause and its health impacts are relevant public health concerns and reveal a need for support to promote adequate self-help strategies, as well as social and professional support (e.g., medical treatment if needed) in order to buffer negative mental health effects. However, many persons are not familiar with the potential impact and treatment options surrounding menopause [[18], [19], [20]], as they believe their symptoms to be (a) part of biological ageing, (b) non-respondent to treatment, or (c) stigmatized and not suited for disclosure. Therefore, easily available and accurate information, positive social norms around disclosure and treatment, and strategies to boost self-help and help-seeking for symptoms can be useful to bridge this gap. To this end, mobile health applications (mHealth apps) are promising to provide accurate and timely health-related information, recovery narratives, build social networks, and establish connections to health professionals more easily. They are easy to access, regardless of physical or time constraints, promise low costs, and relative anonymity [[21], [22], [23]]. Many studies have already examined mHealth apps in similar populations, such as menstrual cycle and/or symptom trackers, which are popular in the premenopausal period [[24], [25], [26]], yet menopause has received considerably less attention so far. In Germany, menopause care is generally provided in primary care or by gynecologists. However, consultations often concentrate on managing acute symptoms rather than offering broader, long-term support for overall well-being. In this context, digital solutions such as smartphone applications may offer an accessible, flexible, and scalable way to support menopausal individuals, particularly when traditional healthcare settings do not fully meet their needs.

In recent years, one study reviewed existing English-language mHealth apps for menopause, and identified 22 apps. Most apps provide information to recognize typical symptoms and learn coping strategies [27]. Further, some apps also support communication through chats and forums, offer monitoring tools (e.g., regarding symptoms), calendars, and information about different available treatment options. However, the quality of most apps was unclear, there were hardly any expert ratings or standardized user ratings available that would allow for evidence-based recommendations. Gkrozou et al. [27] also summarized app store ratings, but did not use validated quality appraisal tools to assess mHealth apps. Quality appraisal of mHealth apps is important, as apps that are not evidence-based may provide insufficient or incorrect information, may prove to be ineffective and may even cause harm (e.g., if they are neglecting to mention possible side effects of treatment options) [28,29]. App store ratings (e.g., stars) are not sufficient indicators of the clinical utility or quality of mHealth apps but rather their popularity [23,30]. Hence, more elaborate, evidence-based rating systems are needed, such as the Mobile Application Rating Scale (MARS) [31], that allows for a multidimensional evaluation regarding the engagement, functionality, aesthetics, and information quality of an mHealth app. It combines user experience and usability research with clinical practice and provides rating scales for medical professionals as well as patients/end-users. Thus, it exceeds global ratings (e.g., stars), and presents a more nuanced perspective on mHealth app quality. The scale has been adapted to the German context [32], psychometrically validated [33], and used to evaluate many different mHealth apps, ranging from depression [33] to sexual health education [34].

Sillence, Hardy, and Kemp [35] combined MARS evaluations with user ratings and reviews of English-language menopause apps, providing important insights into app quality and user experiences. Building on this work, our app review summarizes the current state of mHealth apps for menopause and evaluates their quality using the MARS-G. Unlike previous studies, this review focuses on apps available in German and contributes a distinct perspective by incorporating systematic input from both health experts and users. In doing so, it offers a more comprehensive and context-specific evaluation intended to inform both the German public and health care professionals about available options and their quality.

## 1 Methods

The study was conducted in three steps [1]: searching for and identifying menopause-related mHealth apps in app stores [2], describing the apps and gathering user ratings, and [3] evaluating the quality of the apps by users and health experts (i.e., gynecologists). These steps were guided by the PRISMA 2020 Checklist (see supplementary file).

For the initial phase, we conducted a comprehensive literature review to identify search terms used in meta-analyses, reviews, and databases (e.g., MeSH terms) related to menopause. Additionally, we deployed an anonymous online survey distributed via emails, social media posts, and personal communications to gather insights from medical students, healthcare professionals, and gynecologists. We sought their input on search terms and lay terminology used to explain menopausal issues to the public, as well as their recommended features for menopause apps to enhance app search and selection processes. We also queried their awareness of existing menopause-related apps and whether they had personally used or recommended any.

Another survey was distributed to women aged between 40 and 55 years, aiming to capture a population close to menopause. The survey sought their input on relevant app store search terms related to menopause and their functional needs regarding mHealth apps for menopause. We reached this target group through distribution of leaflets in doctor's offices, online postings in forums dedicated to menopause, and personal contacts.

The subsequent search for menopause apps used a webcrawler which is affiliated with the Mobile Health App Database (MHAD) [36] that searches “Google Play Store” and the Apple “App Store” for mobile applications related to predefined search terms. We used terms related to menopause (e.g., postmenopause) as well as typical symptoms (e.g., hot flashes) to search for apps (see supplementary material for an overview of search terms). Apps eligible for inclusion focused on menopause, were available in German, did not focus on specific drugs or treatments only, and were fully functional when downloaded via the App store. The search and selection took place from June 2021 to August 2021. The apps were tested on an iPhone 12 Mini (model: MGE53QN/A), an iPhone 8 (model: MQ6G2ZD/A) as well as a Samsung Galaxy A12 (model: SM-A125F/DSN), and a Samsung Galaxy Note 10 (model: SMN975F/DS). Two authors performed the search and selection (AP, BS), and they discussed differences with the remaining authors to reach a consensus decision. This process was established in previous reviews of mHealth applications [32,33].

As a second step, we gathered information from app stores for all selected apps. This included details such as name of the app, app store and compatible platform, version (including last update), developer, number and average of user ratings (in-store), app store categories, costs, target group(s), self-reported medical purposes, certification status, data security measures, and scientific evidence base.

In the third step, we invited experts (such as gynecologists and medical professionals specializing in menopause care) as well as menopausal individuals (individuals experiencing pre-, peri-, or postmenopausal symptoms) to test and rate the apps. Each app was rated by at least two independent raters, and ratings were averaged across raters [32,33]. Intraclass correlation was used to describe interrater agreement, a coefficient of > 0.75 indicated sufficient agreement [33]. Ratings were anonymous. We also captured age, gender (and tenure) of raters. Before the rating process, each participant received a leaflet and access to a video-based training explaining the MARS-G and its application. Participants were asked to complete the training and rate an exemplary app before beginning the rating procedure. This training was standardized for all users of the MARS-G and lasts about 30 min in total [32]. Afterwards, participants were invited to download and test the app (they were free to use one of the authors’ devices or their own) for about 10 min, before completing the rating.

### 1.1 Mobile App Rating Scale – German version (MARS-G)

To capture user and expert ratings, we used a German adaptation [19] of the MARS [31]. It consists of 19 items measuring four subscales, namely engagement (5 items; ω = 0.84), functionality (4 items; ω = 0.90), aesthetics (3 items; ω = 0.91), and information quality (7 items; ω = 0.74). Engagement refers to the customizability, and entertainment value of the app, functionality captures its design, usability, and navigation, aesthetics describes the layout, and visual appeal, and information quality helps to qualify and quantify the information given and its accuracy and connection to scientific evidence. All items are rated on a scale from 1 (*inadequate*) to 5 (*excellent*). Items that cannot be applied to an app are rated as not applicable (*n/a*) and are not counted towards the average score. The subscales can be compiled to an average score that indicates overall mHealth app quality in a single index.

Moreover, three additional scales measure the therapeutic gain, subjective quality, and impact on behavior change of the app. Since the MARS was developed with healthcare applications in mind (e.g., psychotherapy), it was adapted for this study to refer to counseling or treatment of persons experiencing menopause-related symptoms. The scale therapeutic gain (four items) measures perceived risks and benefits of using the app as well as side effects and application to routine care. The scale subjective quality (four items) asks participants whether they would personally use or pay for the app. They are also asked to provide a user rating (up to five stars) as a global indicator of quality. Finally, the scale behavior change measures the perceived impact on attitudes, knowledge and intention regarding behavior change following app use (e.g., showing adaptive coping behaviors to deal with hot flashes).

### 1.2 Ethical approval and sampling

The participants were recruited through convenience sampling. Participation was voluntary, and no incentives were provided. All participants gave informed consent to participate in this study. No identifying data were collected to guarantee the anonymity of the participants. At the start of the study, the participants were informed about the purpose of the study, that all data would be processed for research purposes only, that they would remain anonymous, and that they could prematurely stop the study at any point in time. The participants finally indicated informed consent by checking a corresponding box. In Germany, as stated by the German Research Association (Deutsche Forschungsgemeinschaft, DFG), ethics committee approval was not required for this survey because the research did not include a treatment, did not pose any threats or risks to the respondents, and was not associated with high physical or emotional stress; also, the respondents were informed about the objectives of the study before agreeing to participate. The study was in line with ethical standards such as the Declaration of Helsinki and its later amendments or comparable ethical standards as well as national laws (i.e., in alignment with the GDPR) and recommendations of the DFG regarding good scientific practice.

## 2 Results

**Fig. 1:**
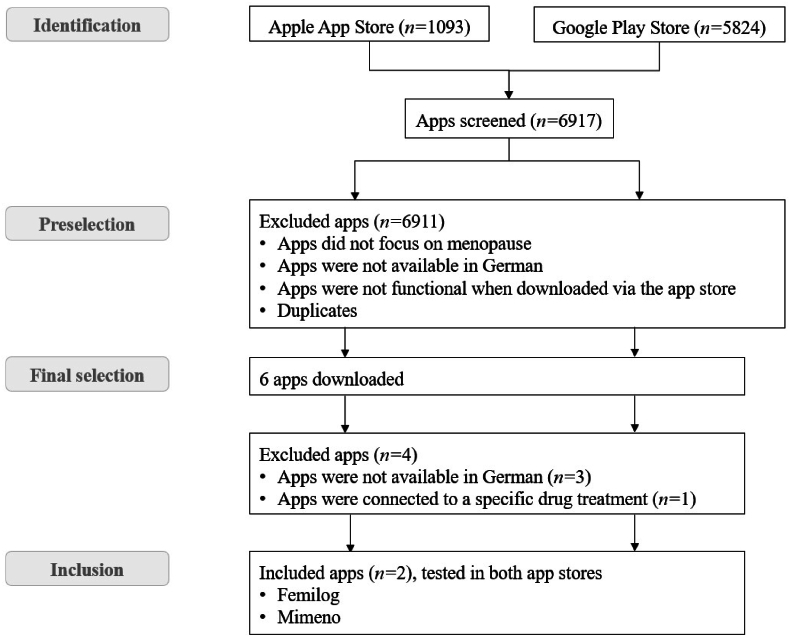
Flow chart of the app selection process*.*

The first survey resulted in *n =* 7 experts (out of 328 contacts) and *n =* 19 women (out of 50 contacts) completing the initial questionnaire, and providing search terms. Combined with the results of the literature review, 32 search terms were used (see supplementary material). The search led to 6917 apps (1093 Apple store, 5824 Google Play store), 6911 were excluded (see Fig. 1 for a flow chart). Six apps were remaining for the final assessment. In the last step, four apps were excluded, because three apps were only available in English, and one app was developed for a specific drug treatment. Two apps were analyzed, both in the iOS and Android version. Survey participants did not know any other apps and reported that they had never used or recommended a mHealth app for menopause to patients before.

The first included app, Femilog, was developed by Femilog ApS, and version 1.0.3–1 (last updated on November 30, 2021) was used for the evaluation. Femilog had received one user rating of 1.0 in the Apple app store and no rating in the Google Play store. It was recommended for users of any age (Google) and 12 years or older (Apple), and listed under *health and fitness apps*. The app was available for a free trial for 14 days, with monthly payments of €6.49 (Apple store) and €5.99 (Google Play store) thereafter. We could not find any certifications, scientific studies or evidence regarding the efficacy or usability of the app. According to its description, the app aims to improve quality of life of persons experiencing menopause by providing a tool to monitor symptoms that can be visualized and shared with physicians or gynecologists. The tool also includes a reminder that supports monitoring, and provides psychoeducation about menopause, symptoms, and coping strategies. The app presents a data security statement that describes data collection and processing and has to be accepted when installing the app.

The second included app, Mimeno, was developed by Medimove GmbH, and version 2.6.3 (last updated on January 1, 2021) was used for the evaluation. Mimeno had received 237 user ratings in the Apple app store, with an average of 4.4 (out of 5), and 18 user ratings in the Google Play store, with an average of 1.6. It was recommended for users of any age (Google) and 17 years or older (Apple), and listed under *health and fitness apps*. The app was available free of charge. We could not find any certifications, scientific studies or evidence regarding the efficacy or usability of the app. According to its description, the app aims to improve quality of life of persons experiencing menopause by providing a tool to monitor symptoms and coping strategies (e.g., taking medication) that can be visualized via monthly graphs. However, it does not provide an interface to share results with physicians or gynecologists to support blended therapy. The tool also includes a reminder that supports monitoring, and provides psychoeducation about menopause, symptoms, and coping strategies. Moreover, it provides access to a private forum that encourages pseudonymized exchange with other app users. The app presents a data security statement that describes data collection and processing and has to be accepted when installing the app.

### 2.1 Quality rating

The apps were rated by four individuals experiencing menopause (all female, 44, 52, 55, and 57 years of age, none of them had used the menopause apps prior to their participation in the study) and two gynecologists (both male, 42 and 67 years of age, with 17 and 37 years of experience in the field). Table 1 presents ratings for all sections of the MARS-G in both groups. In the expert rating, both apps received low ratings (i.e., mean values < 3 on a scale from 1 to 5) for the scales engagement, treatment, and subjective quality. In the user rating, both apps received average scores above 3.0 across all scales. However, Femilog received higher expert ratings than Mimeno, whereas Mimeno received higher user ratings than Femilog. Users were more likely to recommend Mimeno than Femilog, the perceived therapeutic gain was poor for both apps. Interestingly, the subjective quality rating (which includes a five-star rating system, similar to both app stores) had the highest standard deviation among all scales and thus seemed to be least reliable.

**Table 1:** Quality appraisal of menopause apps using the Mobile Application Rating Scale-German (mean values and standard deviations).

MARS-G scales	Femilog		Mimeno	
	Experts	Users	Experts	Users
Engagement	3.30 (0.57)	3.10 (0.64)	2.70 (1.15)	3.80 (0.77)
Functionality	3.75 (0.29)	3.31 (0.60)	2.63 (1.03)	4.38 (0.50)
Aesthetics	4.17 (0.29)	3.33 (0.49)	3.50 (0.50)	4.42 (0.51)
Information Quality	3.08 (0.66)	3.19 (0.40)	2.58 (0.97)	3.63 (0.50)
**Overall (total score)**	3.58 (0.48)	3.23 (0.55)	2.85 (0.43)	4.05 (0.68)
Therapeutic gain	2.63 (0.63)	–	2.75 (0.50)	–
Subjective quality	2.88 (1.49)	2.56 (0.97)	2.25 (1.26)	3.19 (1.05)
Perceived impact	3.08 (0.66)	3.25 (0.44)	2.92 (0.49)	3.71 (0.46)

Interrater agreement (ICC)	0.79	0.76	0.79	0.76

*Notes*. ICC – intraclass correlation; values of MARS-G ratings range from 1 (*inadequate*) to 5 (*excellent*).

## 3 Discussion

This app review evaluates mHealth apps for persons experiencing menopause, and provides expert and user ratings of identified apps, available to the German-speaking population. The review identified two apps that fulfilled all inclusion criteria (Femilog, Mimeno), out of about 7000 records across Apple App store and Google Play store. Both apps received mixed ratings from experts and users, with higher ratings for aesthetics, and lower ratings for information quality or therapeutic gain. This is problematic, as inaccurate knowledge (i.e., low information quality) about phenomena and treatment options in menopause is a major barrier to receiving adequate support [[18], [19], [20]]. Without adequate support, persons can experience a negative impact of menopausal symptoms on their quality of life [[14], [15], [16], [17]].

Interestingly, the apps differed in their ratings from users and experts, although both received moderate appraisals, leaving room for improvement. This suggests that apps may appeal to users due to their design, but their health-related benefits are unclear and real-world evidence is lacking [23,27]. This finding is in line with a study by Ko et al. [24] evaluating the quality of menstrual tracking mobile apps from the perspective of consumers and healthcare providers. The study found that although there were many menstrual tracking apps available in English and Korean, the quality of the apps varied greatly. Consumers and healthcare providers both prioritized accuracy and ease of use in their evaluations of menstrual tracking apps. Consumers placed additional emphasis on usability features such as customization and reminders, while healthcare providers pointed to the need for privacy and security of user data.

Moreover, while one app (Mimeno) has received quite positive app store ratings, the equivalent scale measuring subjective quality showed considerable variance in responses, once more challenging such ratings as a reliable indicator of mHealth app quality in general [23,30] as well as for menopause in particular [23,24,27,30]. Therefore, including a validated rating tool like the MARS-G in addition to App store ratings further stresses the need for evidence-based user-centered development and evaluation of health apps [28,29,31,33]. We were unable to find any certifications, scientific studies or reliable evidence for the two apps meeting the inclusion criteria. Previous reviews also pointed to a lack of scientific evidence regarding app quality [24,27]. Thus, it is important to evaluate mHealth apps further to distinguish between high and low-quality apps. This will enable recommendations to be provided to practitioners and users regarding the most effective and user-friendly apps for managing menopause symptoms. Our study indicates that practitioners were receptive to suggesting apps and incorporating them into their practice. However, there is a deficiency in awareness regarding the existence of high-quality mHealth options.

## 4 Strengths and limitations

The search and selection process for this app review was conducted between June 2021 and August 2021. However, it is possible that new mHealth apps for menopause have been developed since the search was conducted, which may have been missed in this study. While we consider our methodology applicable to newly released apps, future studies should also collect additional information on raters (e.g., menopausal status and medication use), examine usage behavior over time, ensure greater diversity in the study sample, and could further complement quantitative ratings with qualitative feedback.

Moreover, the search yielded only two apps that fulfilled the inclusion criteria. This raises the question of whether the inclusion criteria were too strict, resulting in the exclusion of potentially high-quality apps. Additionally, it could be that there is a lack of mHealth apps specifically designed for menopause, highlighting a need for further development in this area. Other authors also pointed out a lack of apps to support the menopausal phase [39]. Therefore, future studies should review and potentially revise the inclusion criteria to ensure that a wider range of high-quality apps is included in the analysis. A review of English-speaking apps shows a greater variety [27], yet quality of these apps was not thoroughly assessed and many of them were not available in other languages and thus not accessible to a non-English speaking audience. Given the lack of rigorous quality assessment, it would not be appropriate to simply translate or adapt English-speaking apps to other languages (e.g., German), since their quality is not clear.

In addition, we focused on apps for persons undergoing a natural and/or typical menopausal transition linked with hormonal changes. This excludes specific groups such as persons trying to achieve pregnancy despite early menopause onset [40] or persons with early onset menopause [5] as well as persons living with multiple health conditions, such as menopause-related symptoms and obesity [41]. It is possible that these groups have unique needs or questions that are not addressed by apps that provide information on common experiences and coping strategies but that are not tailored to their needs. Therefore, future studies could explore mHealth support for these groups.

In contrast to a mere quality rating from a researcher's perspective, as conducted in comparable studies [35,37,42], we consulted users and practitioners from the field who gave their evaluation. Even though we have had very small samples, we see this user-expert-involvement as a clear strength of our method. Finally, we followed established protocols for expert and user ratings of the apps [33], yet future studies could provide real-world evidence of mHealth app use and quality ratings in everyday life, for instance, via ambulatory assessments. When interpreting the results, it is important to acknowledge the potential differences between app evaluation using the Mobile App Rating Scale (MARS) and actual long-term use in real-world contexts. The MARS focuses on structured assessments of app quality across domains such as engagement, functionality, aesthetics, and information quality. While this provides a systematic and standardized snapshot of app quality at a single point in time, it does not fully capture the dynamics of sustained use, user engagement, and effectiveness in everyday practice. In longer-term use, factors such as novelty effects, user fatigue, and contextual fit (e.g., how well the app integrates into a user's daily routines and personal goals) may alter perceptions of quality and actual impact. However, this would require a more elaborate, longitudinal study design and thus more resources.

Some of the analyzed apps may no longer be available for download due to the rapidly changing app market. Other studies have highlighted this limitation inherent in the field of app research as well [37,38]. Prior to the publication of this article, Femilog was still available for download, whereas Mimeno was no longer accessible.

## 5 Implications for practioners

The results of our study indicate that practitioners should not recommend mHealth apps for menopause without reservation. It is essential for them to critically evaluate the apps their (peri)menopausal individuals use or are considering for support. Familiarity with the MARS-G criteria can assist practitioners in efficiently determining whether a potentially relevant app meets scientific standards [31,32]. Moreover, menopausal individuals also need to know what to expect and how to use the app, so it is recommended that practitioners and users go through the app together to assess its potential and utility for self-help and the treatment process.

## 6 Conclusion

In conclusion, while this app review offers a valuable starting point for evaluating mHealth apps for menopause in German, much work remains to fully understand the landscape of available apps and their efficacy and usability. Future studies should consider a broader time frame and more flexible inclusion criteria, and they should evaluate the efficacy and usability of identified apps to provide evidence-based recommendations for practitioners and users.

## CRediT authorship contribution statement

**Klara Greffin:** Writing – review & editing, Writing – original draft, Visualization, Validation, Supervision, Resources, Project administration, Methodology, Investigation, Data curation, Conceptualization. **Marlene Muehlmann:** Writing – review & editing, Validation, Software, Methodology. **Anna Pauleikhoff:** Writing – original draft, Validation, Software, Methodology, Formal analysis, Data curation. **Bita Stelling:** Writing – original draft, Validation, Software, Methodology, Formal analysis, Data curation. **Holger Muehlan:** Writing – review & editing, Resources. **Michael Stach:** Writing – review & editing, Software. **Silke Schmidt:** Writing – review & editing, Supervision. **Samuel Tomczyk:** Writing – original draft, Visualization, Validation, Supervision, Resources, Project administration, Methodology, Investigation, Formal analysis, Data curation, Conceptualization.

## Ethics approval and consent to participate

Ethics approval: Not applicable. Consent to participate: All participants gave informed consent to participate in this study.

## Consent for publication

Not applicable.

## Availability of data and materials

The data that support the findings of this study are available upon request. Therefore, please contact Samuel Tomczyk (samuel.tomczyk@uni-greifswald.de).

## Funding

This study did not receive any external funding.

## Declaration of competing interest

The authors state that they have no conflicts of interest.
