# Supplementary material for: Embracing change: Navigating menopause with the help of mobile health apps in Germany
Source: Compr Psychoneuroendocrinol. 2025 Sep 25;24:100320. doi: 10.1016/j.cpnec.2025.100320 (PMC12519169; doi:10.1016/j.cpnec.2025.100320)
Supplement: Multimedia component 1 [file mmc1.docx]

**Supplementary Material**

Search terms

Menopause, menopause, Wechseljahre, Schlafstörung, Schlaflosigkeit, Hitzewallung, Wechseljahresbeschwerden, Östrogen, Gelenkschmerzen, Gewichtszunahme, Postmenopause, Stimmungsschwankung, schwitzen, Depression, Gestagen, Müdigkeit, Schweißausbrüche, Lustlosigkeit, Libidoverlust, Klimakterium, Hormontherapie, Klimax, Hormone, Naturheilverfahren, sleep disorder, Blutungsstörung, hot flushes, climax, Scheidentrockenheit, Schleimhauttrockenheit, starke Regelblutung und Sex.
